# Supplementary material for: Hypergraph reconstruction from dynamics
Source: Nat Commun. 2025 Mar 19;16:2691. doi: 10.1038/s41467-025-57664-2 (PMC11923283; doi:10.1038/s41467-025-57664-2)
Supplement: Supplementary file 1 — Supplementary information [file 41467_2025_57664_MOESM1_ESM.pdf]

Supplementary Information

Hypergraph reconstruction from dynamics

Robin Delabays, Giulia De Pasquale, Florian Dörfler, and Yuanzhao Zhang

S1. NETWORK INFERENCE, ARNI VS. THIS

Here, we compare ARNI [1] and THIS using synthetic data generated by Kuramoto oscillators on random graphs. The ROC curves are shown in Supplementary Fig. 1 for THIS (top left) and ARNI with five different function bases. The different curves are colored according to the length of the time series used (the darker the shorter). We see that with suitable basis functions, ARNI can perform as well as THIS, but a non-ideal choice of basis functions significantly reduces ARNI's performance.

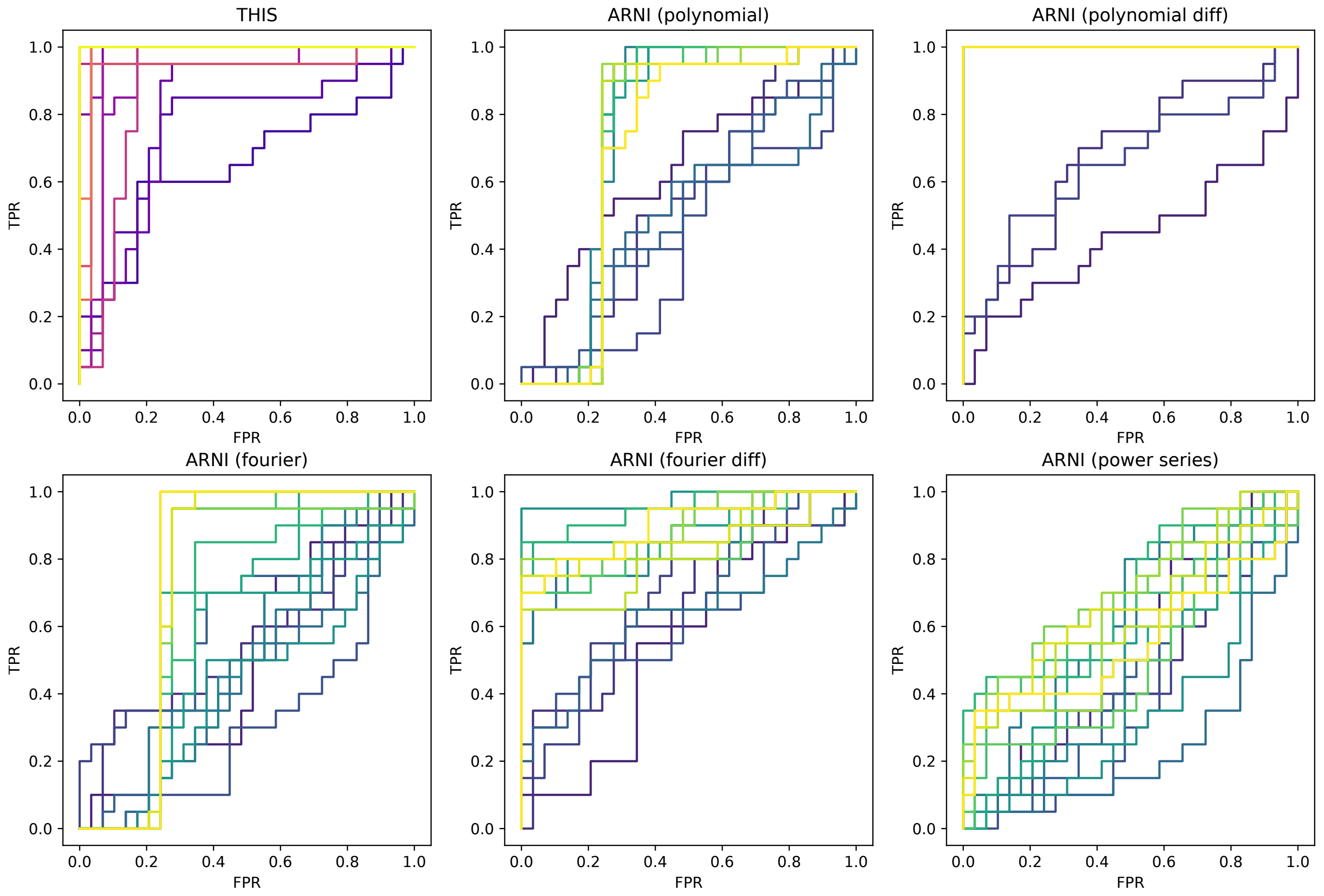

Supplementary Figure 1. **Comparison between ARNI and THIS on the task of inferring pairwise interactions.** Each panel shows the ROC curves for the inference of the adjacency matrix, based on a time series generated by seven Kuramoto oscillators coupled through an Erdös-Rényi random graph ($p = 0.5$). The same time series are used in each panel. Each curve corresponds to a different length of the time series (the darker the shorter), ranging from 8 to 80 data points. Each point is sampled uniformly from a box of size 1.5 (radians) centered at the origin and the exact time derivative is computed from the ODE at each time point. The top left panel is our method, the others are ARNI with the five main function bases proposed in Ref. [1]. Namely, polynomial basis, polynomial basis of differences, Fourier basis, Fourier basis of differences, and power series basis (see [1] for details). Our method achieves similar performance as the best version of ARNI, but much better than ARNI with a non-ideal basis (there is no way to know which basis is better a priori when using ARNI). Moreover, while we focused on inferring pairwise interactions here when using ARNI, we made the task harder for THIS by allowing it to infer third-order interactions as well.

S2. TUNING THE BOX SIZE

As mentioned in the main text, THIS requires the time series to be sampled in a domain large enough to sufficiently explore the dynamics, but not too large so as to preserve the accuracy of the Taylor approximation. In Supplementary Fig. 2, we show the AUC of the inference as a function of the size of the sampling domain. We observe that there is a wide range of box sizes for which THIS performs well. For the sake of comparison, we tested ARNI with the same data and found that the box size does not influence its performance.

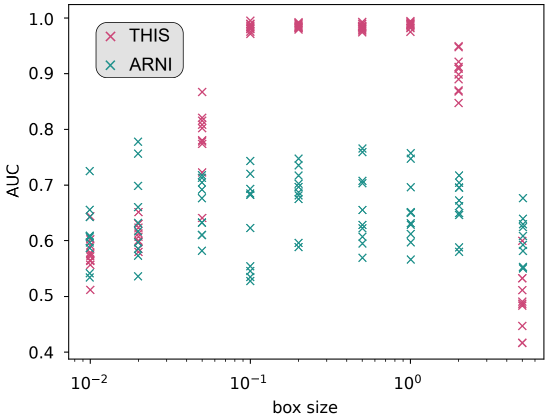

Supplementary Figure 2. **Quality of the inference with respect to the size of the sampling area.** The AUC is the area under the ROC curve for the inference (1 means perfect inference while 0.5 means performance comparable to random guesses). For each box size, we generate ten hypergraphs with Kuramoto-like dynamics. For each test, the inference is performed with 100 initial conditions and perfect measurement of the time derivatives. For THIS (pink), we see a trade-off between a box that is too small to accurately represent the dynamics and a box that is too large to admit a faithful Taylor approximation. For ARNI (green), the box size does not influence its performance.

S3. ALGORITHMIC COMPLEXITY

Supplementary Figure 3 shows a polynomial growth of the computational cost of THIS (including pre-filtering) with respect to the number of nodes. A rough slope estimate suggests that the computation time grows approximately as n^4. Notice that the inference was not parallelized here. If THIS were run on n CPUs, the computation time would scale as n^3.

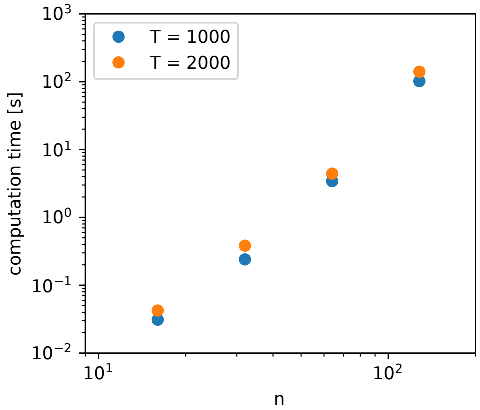

Supplementary Figure 3. **Computation time as a function of the system size.** We ran THIS on time series of length $T = 1000$ (blue) and $T = 2000$ (orange) for Kuramoto dynamics on sparse simplicial complexes with both second-order and third-order interactions. For each system size, the computation time is averaged over 5 different random simplicial complexes. A pre-filtering step was performed, keeping 10% of the most correlated pairs.

S4. BREAKDOWN OF ROC CURVES

In Supplementary Fig. 4, we show the ROC curves from Fig. 2 in the main text (left panels), and split each curve according to pairwise interactions (middle panels) and 3-body interactions (right panels).

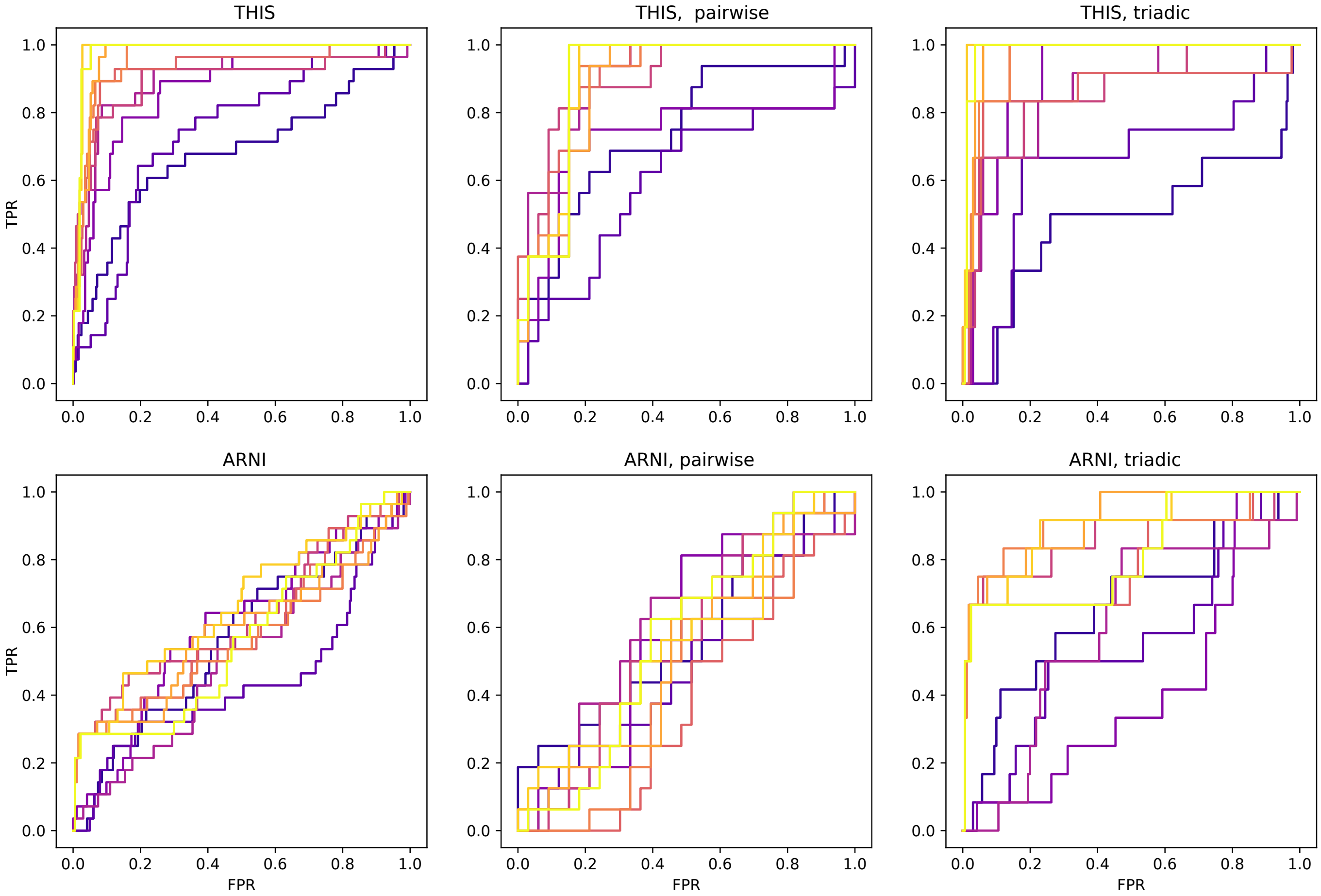

Supplementary Figure 4. **ROC curves for THIS (top row) and ARNI (bottom row).** The left panels repeat the ROC curves of Fig. 2 from the main text, i.e., the ROC curves for the inference of all (hyper)edges. The middle (right) panels show the ROC curves for the inference of the pairwise (triadic) interactions only, while inferring both second- and third-order interactions. Each curve corresponds to a different sample size used in inference, ranging from 10 data points (dark purple) to 150 data points (bright yellow).

S5. RESULTS FOR LARGE RANDOM SIMPLICIAL COMPLEXES AND HYPERGRAPHS

Supplementary Figure 5 reproduces Fig. 3 from the main text (left panel) and further breaks down the ROC curves according to pairwise (middle panel) and triadic (right panel) interactions. Supplementary Figure 6 shows similar results for a 100-node random hypergraph.

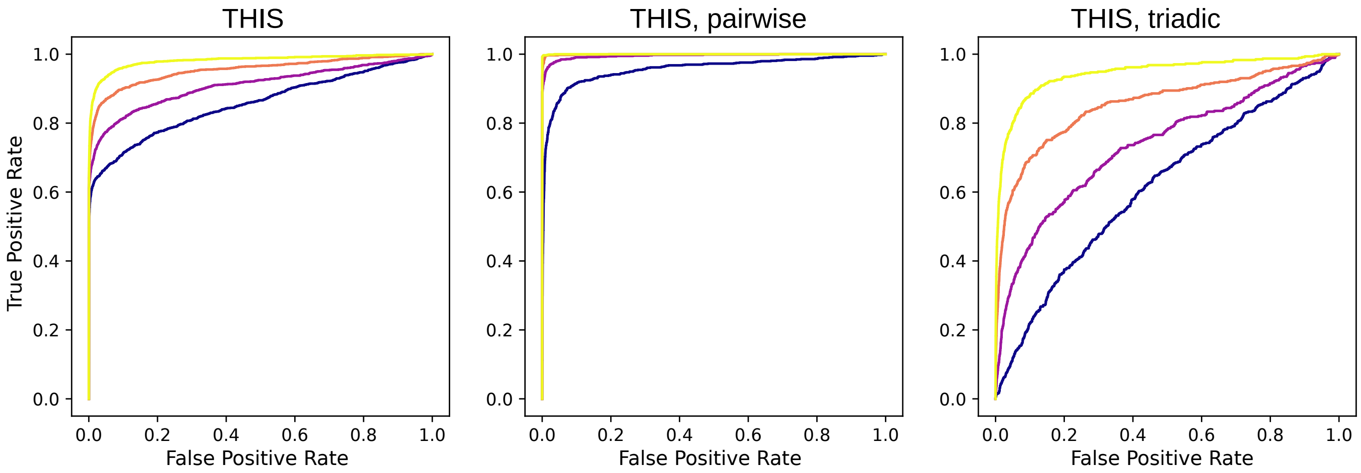

Supplementary Figure 5. **Inferring a 100-node simplicial complex from generalized Kuramoto dynamics.** The middle and right panels are the ROC curves for the pairwise and triadic interactions, respectively. The left panel repeats Fig. 3 from the main text for completeness.

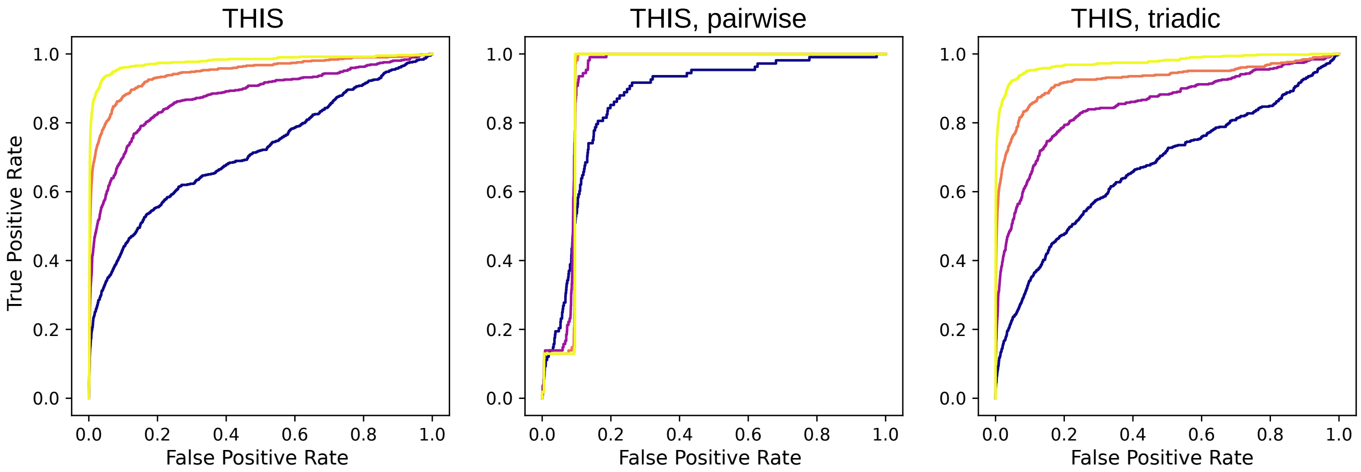

Supplementary Figure 6. **Inferring a 100-node random hypergraph from generalized Kuramoto dynamics.** We show the hypergraph analog of Fig. 3 from the main text. The hypergraph is randomly generated by an Erdős-Rényi-type process, where each 2-edge (resp. 3-edge) exists with probability 1% (resp. 0.1%). Each color corresponds to a different number of data points, increasing from 500 (dark blue) to 2000 (bright yellow).

S6. SENSOR PLACEMENT AND SIGNAL AGGREGATION

Supplementary Figure 7 shows the location of 64 sensors on the scalp, and their aggregation into seven groups, inside which the time series were averaged before the inference.

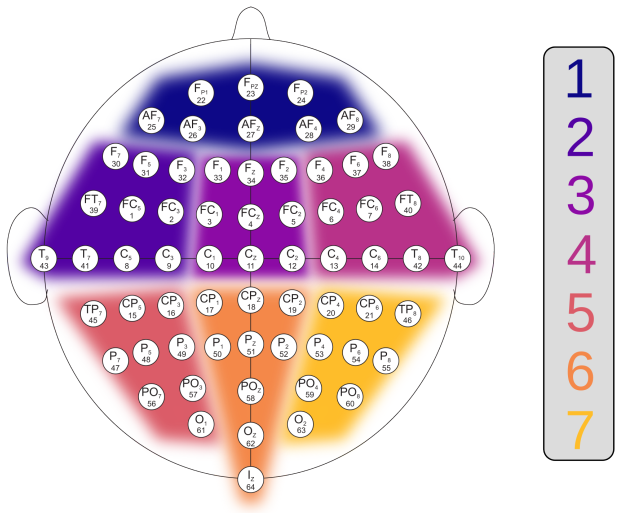

Supplementary Figure 7. **Location of the 64 sensors for the EEG measurements.** The colors indicate the seven aggregation areas used in our analysis.

S7. FITTING ERROR VERSUS INTERACTION ORDER

Supplementary Figure 8 shows the relative fitting error of the inference algorithm as different orders of interactions are included, up to the seventh order (the maximum possible). We see that including interactions up to the fourth order achieves a good balance between accuracy and parsimony—with a median fitting error of around 0.3, it is able to capture the overall behavior of the EEG data without overfitting the noise. Thus, we restrict our attention to interactions up to the fourth order in our analyses.

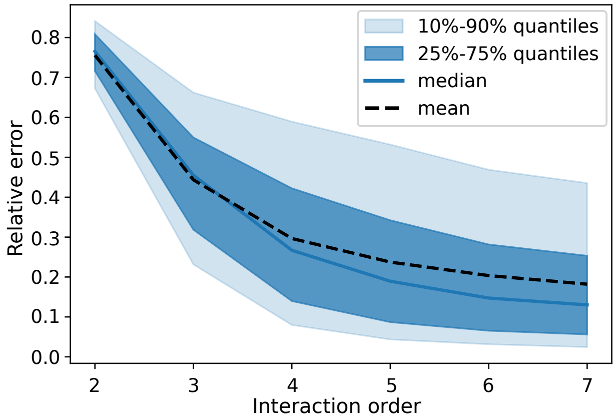

Supplementary Figure 8. **Statistics of the relative fitting error on the coarse-grained EEG data, as more orders of interactions are included.** The relative error is computed as the absolute difference between the inferred time series and the measured time series, normalized by the absolute value of the measured time series. We can see that interactions up to the fourth order are the ones that contribute the most to minimizing the fitting error. Including interactions beyond the fourth order only brings marginal improvements and increases the risk of overfitting.

S8. RELATIVE CONTRIBUTIONS FROM EACH ORDER OF INTERACTIONS

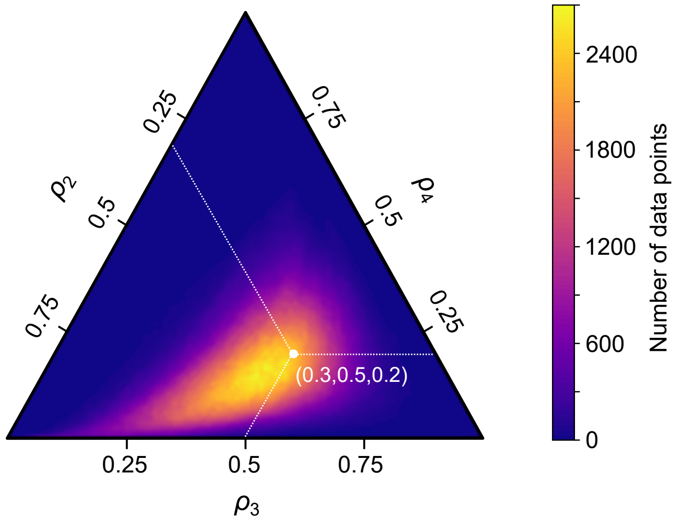

Supplementary Figure 9. **Relative contribution from the second-, third-, and fourth-order interactions to brain dynamics based on the coarse-grained EEG data.** The colors indicate how often a combination of contribution ratios occur (they all add up to 1, see equation (4) in the main text). We see that third-order interactions explain the most amount of dynamics at around 45%, pairwise interactions explain about 35%, and fourth-order interactions explain the remaining 20% of the dynamics. The dotted lines serve as a guide to read the figure.

S9. ANALYSIS OF THE FULL 64-CHANNEL EEG DATA

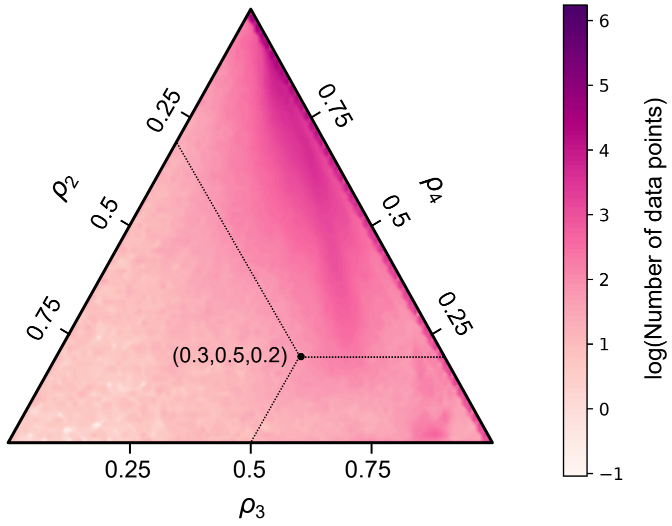

Supplementary Figure 10. **Relative contribution of the second-, third-, and fourth-order interactions to brain dynamics based on the full 64-channel EEG data.** Similar to the case of coarse-grained EEG data, nonpairwise interactions contribute significantly to the dynamics. The dotted lines serve as a guide to read the figure.

[1] Casadiego, J., Nitzan, M., Hallerberg, S. & Timme, M. Model-free inference of direct network interactions from nonlinear collective dynamics. *Nature Communications* **8**, 2192 (2017).
